# 

**Published:** 1866-02

**Authors:** 


### The Chicago Medical Journal.

CONTENTS FOR FEBRUARY.

ORIGINAL CONTRIBUTIONS.

**E. L. HOLMES, M. D.,**

**H. M. LYMAN, M. D.,**

**R. M. LACKEY, M. D.,** } *Editors.*

A MONTHLY JOURNAL

DEVOTED TO THE CULTURE OF MEDICAL AND SURGICAL SCIENCE.

It will be our aim to secure for our readers original papers on important medical subjects; to present valuable selections from foreign journals, and from our large exchange list of American medical periodicals; to obtain reports of interesting cases, observed in the Hospitals, Infirmaries and Dispensaries of Chicago, and to call the attention of the Profession to new and valuable works in the various departments of medical science.

While it will be our chief object to aid in promoting the best interests of the Profession in the culture of true science, we shall also carefully endeavor to publish such intelligence, not strictly scientific, as may be of particular interest to the practising physicians of the Northwest.

All letters for the JOURNAL should be addressed to R. M. LACKEY, M.D., P. O. Box 2175, Chicago, Ill. *Office—169 South Dearborn Street.*

TERMS—$2 PER ANNUM, IN ADVANCE.

$3 if not paid before 1st of July of each year. Single Copies, 25 cents.

### THE AMERICAN MEDICAL ASSOCIATION

Will hold its annual meeting at Baltimore on the first Tuesday of May next.

The meeting at Boston last year was unusally large, and proved highly profitable as well as pleasant to all in attendance. A full meeting may be expected this year at Baltimore. The profession of the Northwest should be well represented.

The character of the members appointed on the different committees, will undoubtedly ensure most valuable papers on the various subjects selected for special reports.

Since the annual meeting of the Illinois Medical Society will take place at Decatur, on the first Tuesday in June next, one month after that of the National Association at Baltimore, it will be necessary for the delegates of the State Society, appointed at Bloomington last year, to the National Association, to act also this year as delegates to the coming Convention at Baltimore.

The following are the names of the delegates: A. L. McArthur, M. D., of Joliet; L. T. Hewins, M. D., of Loda; J. W. Redden, M. D., of Shawneetown; J. M. Steele, M. D., of Grandview; J. S. Jewell, M. D., of Chicago; H. Noble, M. D., of Heyworth; T. F. Worrell, M. D., of Bloomington; E. L. Holmes, M. D., of Chicago; J. W. Freer, M. D., of Chicago; E. W. Moore, M. D., of Decatur; A. Niles, M. D., of Quincy; A. W. Heise, M. D., of Joliet; R. E. McVey, M. D., of Waverly; L. Clark, M. D., of Rockford; H. H. Luce, M. D., of Bloomington; D. Prince, M. D., of Jacksonville; D. Brainard, M. D., of Chicago; W. A. Elder, M. D., of Bloomington; J. Brown, M. D., of Decatur; N. Wright, M. D., of Chatham.

---

### THE ILLINOIS STATE MEDICAL SOCIETY.

The regular annual meeting of this Association will be held at Decatur, on the first Tuesday on June next. It should be remembered that the meeting is appointed one month later than usual, to give members of the Society an opportunity of also attending the meeting of the National Association at Baltimore, on the first of May.

It is perhaps useless to urge upon the profession of Illinois a large attendance upon the meeting at Decatur, for past experience has shown that comparatively few physicians take sufficient interest either to prepare original contributions for the Society, or even to be present at its meeting. The practising physicians of Illinois should not forget, however, that there are advantages in such an organization, which they all, directly or indirectly, might secure.

For one, we believe the Society, even in its present condition, accomplishes a good which is felt throughout the State, although most practitioners might scarcely be conscious of it. We believe also, that it is the small beginning of an organization which in the future is to exert a great and good influence not only on the profession but on the people of the States.

The interests of the profession are certainly advanced by the local, as well as by the State and National Associations. With all their imperfections, they tend to advance medical education by encouraging writing and discussion, and to strengthen the efforts of the profession in carrying forward reforms in hygiene, quarantine, registration of births, marriages and deaths, and in preventing immoral advertisements. A persistent, well directed effort on the part of a State Medical Society, composed of judicious delegates from every county and large town, could ensure in the State Legislature the passage of almost any act really important for the welfare of the people.

If our counties fail to accomplish all that is desirable, there is so much the reason why members of our profession, instead of simply finding fault with their imperfections, should make more combined effort to improve their character and increase their influence, if not by original contributions, at least by their presence and encouragement.

This could be done with but a small tax upon the time and energies of each individual, although organization cannot be extended to banish all ignorance and prejudice from our people, or even from our own ranks, or to wholly counteract the efforts of charlatanry, it can aid in placing medical science higher in the estimation of the public, and teach the people to better appreciate the councils of enlightened physicians in public measures to prevent and overcome disease.

